# Genetic Analysis Reveals the Prognostic Significance of the DNA Mismatch Repair Gene *MSH2* in Advanced Prostate Cancer

**DOI:** 10.3390/cancers14010223

**Published:** 2022-01-04

**Authors:** Hao-Han Chang, Cheng-Hsueh Lee, Yei-Tsung Chen, Chao-Yuan Huang, Chia-Cheng Yu, Victor C. Lin, Jiun-Hung Geng, Te-Ling Lu, Shu-Pin Huang, Bo-Ying Bao

**Affiliations:** 1 Department of Urology, Kaohsiung Medical University Hospital, Kaohsiung 807, Taiwan; 1060503@kmuh.org.tw (H.-H.C.); 1000660@kmuh.org.tw (C.-H.L.); 1000596@kmuh.org.tw (J.-H.G.); 2 Graduate Institute of Clinical Medicine, College of Medicine, Kaohsiung Medical University, Kaohsiung 807, Taiwan; 3 Department of Life Sciences and Institute of Genome Sciences, National Yang Ming Chiao Tung University, Taipei 112, Taiwan; yeitsungchen@ym.edu.tw; 4 Department of Urology, College of Medicine, National Taiwan University Hospital, National Taiwan University, Taipei 100, Taiwan; cyhuang0909@ntu.edu.tw; 5 Division of Urology, Department of Surgery, Kaohsiung Veterans General Hospital, Kaohsiung 813, Taiwan; ccyu@vghks.gov.tw; 6 Department of Urology, School of Medicine, National Yang-Ming University, Taipei 112, Taiwan; 7 Department of Pharmacy, Tajen University, Pingtung 907, Taiwan; 8 Department of Urology, E-Da Hospital, Kaohsiung 824, Taiwan; ed102161@edah.org.tw; 9 School of Medicine for International Students, I-Shou University, Kaohsiung 840, Taiwan; 10 Department of Urology, Kaohsiung Municipal Hsiao-Kang Hospital, Kaohsiung 812, Taiwan; 11 Department of Pharmacy, China Medical University, Taichung 404, Taiwan; lutl@mail.cmu.edu.tw; 12 Department of Urology, Faculty of Medicine, College of Medicine, Kaohsiung Medical University, Kaohsiung 807, Taiwan; 13 Ph.D. Program in Environmental and Occupational Medicine, College of Medicine, Kaohsiung Medical University, Kaohsiung 807, Taiwan; 14 Sex Hormone Research Center, China Medical University Hospital, Taichung 404, Taiwan; 15 Department of Nursing, Asia University, Taichung 413, Taiwan

**Keywords:** DNA damage repair, prostate cancer, survival, single nucleotide polymorphisms, *MSH2*

## Abstract

**Simple Summary:**

Androgen deprivation therapy is the most effective and widely used treatment for advanced prostate cancer, but its efficacy is highly variable among patients. Therefore, the identification of potent prognostic biomarkers is needed to determine patients at risk. We demonstrated that *MSH2* rs1400633 was notably associated with patient survival during androgen deprivation therapy even after adjustment for clinical predictors and false discovery rate correction. Furthermore, our meta-analyses demonstrated that the *MSH2* gene is highly expressed in prostate cancer and correlates positively with poor prognosis for this disease.

**Abstract:**

DNA damage repair is frequently dysregulated in advanced prostate cancer and has been linked to cancer susceptibility and survival outcomes. The aim of this study is to assess the influence of genetic variants in DNA damage repair pathways on the prognosis of prostate cancer. Specifically, 167 single nucleotide polymorphisms (SNPs) in 18 DNA damage repair pathway genes were assessed for association with cancer-specific survival (CSS), overall survival (OS), and progression-free survival (PFS) in a cohort of 630 patients with advanced prostate cancer receiving androgen deprivation therapy. Univariate analysis identified four SNPs associated with CSS, four with OS, and two with PFS. However, only *MSH2* rs1400633 C > G showed a significant association upon multivariate analysis and multiple testing adjustments (hazard ratio = 0.75, 95% confidence interval = 0.63–0.90, *p* = 0.002). Furthermore, rs1400633 risk allele C increased *MSH2* expression in the prostate and other tissues, which correlated with more aggressive prostate cancer characteristics. A meta-analysis of 31 gene expression datasets revealed significantly higher *MSH2* expression in prostate cancer than in normal tissues (*p* < 0.001), and this high expression was associated with a poor prognosis of prostate cancer (*p* = 0.002). In summary, we identified *MSH2* rs1400633 as an independent prognostic biomarker for prostate cancer survival, and the association of *MSH2* with cancer progression lends relevance to our findings.

## 1. Introduction

Prostate cancer is one of the most common cancers in men. Therefore, preventing, diagnosing, and treating prostate cancer is an important public health issue. Currently, the known risk factors for prostate cancer are age, ethnicity, inherited genetic variants, and dietary factors. The risk of developing prostate cancer increases with age, particularly after age 50. It has been reported that age-adjusted prostate cancer incidence rates per 100,000 men vary substantially among ethnic groups, with 647 for African-Caribbeans, 213 for Europeans, and 199 for Asians [1]. These differences are partly explained by the findings that prostate cancer is highly heritable, with an estimated heritability of 58% in the Nordic Twin studies [2]. A variety of dietary factors have also been implicated with respect to prostate cancer risk. Considerable evidence has demonstrated that dairy intake is positively associated with the risk of prostate cancer [3], and cooked tomato/lycopene intake is inversely associated with the risk of advanced disease [4]. The incidence of prostate cancer in Taiwan has increased rapidly over the past decades, and it has become the fifth most common cancer with an age-adjusted incidence rate of 31.65 per 100,000 men in 2017. Following the introduction of prostate-specific antigen (PSA) screening, most prostate cancers are diagnosed at curative early stages, but approximately 10% are diagnosed at an already advanced stage [5]. Prostate cancer is a hormone-dependent cancer whereby androgen signalling plays a pivotal role in both normal prostate development and the pathogenesis of prostate cancer [6]. Therefore, androgen deprivation therapy (ADT) has become the main therapeutic approach against advanced prostate cancer. Although most prostate cancers initially respond to ADT, they gradually develop resistance and progress to castration-resistant prostate cancer, which eventually becomes lethal. Several clinical factors, such as PSA level, tumour stage, and Gleason score, can predict the outcomes of prostate cancer. However, the progression and survival during ADT still differ significantly among patients with similar disease characteristics.

The dysregulation of genes involved in DNA damage repair (DDR) pathways can lead to higher mutation rates and genomic instability, which are common drivers of tumorigenesis in prostate cancer [7]. A study used the comet assay to demonstrate that patients with prostate cancer exhibited higher DNA damage in peripheral blood lymphocytes [8]. It has also been reported that oxidative DNA damage in patients with prostate cancer, based on the measurement of urinary 8-hydroxy-2′-deoxyguanosine, is significantly higher than in healthy controls, while ADT is able to reduce the damage levels [9]. Eukaryotic cells have evolved five major DDR pathways aimed at recognising DNA damage, activating cell cycle checkpoints, initiating DNA repair, and maintaining genomic integrity. Base excision repair removes the small base adducts resulting from alkylating agents, ionizing radiation, and reactive oxygen species, whereas nucleotide excision repair removes DNA crosslinks and bulky lesions resulting from ultraviolet radiation and chemical mutagens [10]. The mismatch repair gene removes the base mismatches originating during DNA replication and recombination. DNA double-strand breaks are repaired by either homologous recombination, an error-free pathway that uses a homologous template to repair the DNA, or non-homologous end joining, an error-prone pathway that ligates the broken ends directly. DDR gene alterations have been found to be more common in advanced/metastatic than localized prostate tumours [11,12]. Among advanced prostate cancers, 60% have clinically actionable genetic alterations in non-androgen-related pathways, particularly in the DDR genes [13]. The most frequently mutated genes were homologous recombination genes, such as *BRCA1*, *BRCA2*, and *ATM*, and mismatch repair genes, such as *MSH2* and *MLH1* [12,13]. However, there are limited immunohistochemical analyses of DDR proteins that have been performed in prostate cancer clinical specimens, and the results are largely inconclusive. One cohort study on prostate cancer specimens from 80 patients demonstrated that the BRCA2 protein is significantly lost in carcinoma cells (77%) compared to normal and hyperplastic prostate tissues (20%) [14]. Conversely, a study with 510 cases found that the BRCA2 protein was overexpressed in 41.6% of prostate tumours and was correlated with poor biochemical recurrence-free survival [15]. Association studies have suggested that the genetic variants in DDR pathway genes may predispose individuals to prostate cancer onset [16,17], tumour aggressiveness [18,19], and treatment outcomes [20,21]. Variants in *XRCC1*, *ERCC2*, *EXO1*, and *MSH6* have been shown to be associated with an altered risk of toxicity, disease recurrence, and survival after treatment for prostate cancer [22,23,24]. Identification of specific biomarkers that may predict the risk of aggressive disease would likely improve the clinical utility of genetic testing to offer patients with an effective and personalised therapeutic plan.

Genome-wide association studies have revealed more than 120 single nucleotide polymorphisms (SNPs) in multiple chromosomal regions, including 8q24, 17q12, and 17q24, associated with a hereditary predisposition to prostate cancer [25]. Potentially, functional SNPs of various genes involved in cancer progression could also determine the efficacy of ADT. Therefore, we comprehensively evaluated the associations of SNPs in major DDR pathway genes with prostate cancer survival in men treated with ADT. We also assessed the potential of the candidate variant/gene as a prognostic biomarker for prostate cancer.

## 2. Materials and Methods

### 2.1. Patients and Response Evaluation

A total of 630 patients who underwent ADT (orchiectomy or luteinizing hormone–releasing hormone with or without an antiandrogen) for advanced prostate cancer were recruited from the following three medical centres in Taiwan: National Taiwan University Hospital, Kaohsiung Medical University Hospital, and Kaohsiung Veterans General Hospital, as described previously [26,27]. Clinicopathological information was extracted from patient medical records. Patients were followed for survival outcomes from the initiation of ADT until 31 August 2020, the date of disease progression (progression-free survival, PFS), or the date of death due to cancer (cancer-specific survival, CSS) or any other disease (overall survival, OS) by linking the personal identification number with the National Death Registry via the Ministry of Health and Welfare, Taiwan. After signing an informed consent form, all participants underwent a structured face-to-face interview and provided blood samples for DNA extraction and genotyping.

### 2.2. SNP Selection and Genotyping

A literature review yielded a list of 18 commonly altered genes in DDR pathways [28], including ATM serine/threonine kinase (*ATM*), ATR serine/threonine kinase (*ATR*), BRCA1 DNA repair associated (*BRCA1*), BRCA2 DNA repair associated (*BRCA2*), BRCA1-interacting helicase 1 (*BRIP1*), checkpoint kinase 2 (*CHEK2*), abraxas 1 BRCA1-A complex subunit (*ABRAXAS1*), GEN1 Holliday junction 5′ flap endonuclease (*GEN1*), mutL homolog 1 (*MLH1*), MRE11 homolog double-strand break repair nuclease (*MRE11*), mutS homolog 2 (*MSH2*), mutS homolog 6 (*MSH6*), nibrin (*NBN*), partner and localizer of BRCA2 (*PALB2*), PMS1 homolog 2 mismatch repair system component (*PMS2*), RAD51 paralog C (*RAD51C*), RAD51 paralog D (*RAD51D*), and X-ray repair cross-complementing 2 (*XRCC2*). Haplotype tagging SNPs (htSNPs) within these genes and 10-kb flanking regions were selected by pairwise linkage disequilibrium with *r*^2^ > 0.8 and minor allele frequency >0.05 using the 1000 Genomes data for Han Chinese in Beijing, China, and Southern Han Chinese data [29] with tagger algorithm [30]. Genomic DNA was isolated from 3 mL of whole peripheral blood using the QIAamp DNA Blood Maxi Kit (Qiagen, Valencia, CA, USA) in accordance with manufacturer’s instructions, and genotyped using the Affymetrix Axiom genotyping arrays system (Thermo Fisher Scientific, Waltham, MA, USA) at the National Centre for Genome Medicine, Taiwan, as described previously [31]. Any SNP that failed in >10% of the samples, had minor allele frequency <0.05, and exhibited a deviation from Hardy–Weinberg equilibrium <0.001 was excluded. Finally, 167 htSNPs were kept for further exploration.

### 2.3. Bioinformatics Analysis

HaploReg v4.1 was used to predict the potential regulatory function of *MSH2* rs1400633 [32]. Expression quantitative trait loci analysis was used to investigate the correlation between rs1400633 and *MSH2* levels based on Genotypes-Tissue Expression (GTEx) data [33]. All publicly available prostate gene expression datasets in The Cancer Genome Atlas Prostate Adenocarcinoma (TCGA PRAD) [34], Oncomine [35], and Gene Expression database of Normal and Tumor tissues 2 [36] databases were used to compare *MSH2* levels between tumour and normal tissues, tumour characteristics, and patient prognoses.

### 2.4. Statistical Analysis

Univariate Cox regressions and log-rank tests were employed to compare differences in CSS, OS, and PFS among patients with diverse clinicopathological characteristics and SNP genotypes. Multivariate Cox regression was further performed to adjust the associations for age, stage, Gleason score at diagnosis, PSA at ADT initiation, PSA nadir, and time to PSA nadir. The false discovery rate (*q* Value) was used to adjust for multiple testing. The link between gene expression and tumour characteristics was assessed by Spearman correlation. Pooled standardised mean differences were used to assess the difference in gene expression between tumour and normal tissues, and the pooled hazard ratio (HR) was used to evaluate the relationship between gene expression and survival outcomes. If heterogeneity was absent from the included studies, a fixed-effect model was used to calculate pooled data; otherwise, the random-effect model was taken into account using RevMan 5.4.1 (Cochrane, London, UK). Other statistical analyses were conducted using Statistical Product and Service Solutions version 19.0.0 (IBM, Armonk, NY, USA). A two-sided *p* Value < 0.05 was considered significant.

## 3. Results

The present study included 630 patients who underwent ADT for prostate cancer, and whose characteristics are presented in Table 1. During a median follow-up of 165.8 months, 314 (49.8%) patients died from cancer, 414 (65.7%) died from another disease, and 518 (82.6%) exhibited disease progression. Age at diagnosis was only associated with OS and PFS. Five other clinicopathological covariates, including PSA, clinical stage, Gleason score at diagnosis, PSA nadir, and time to PSA nadir, were all significant predictors of CSS, OS, and PFS during ADT.

**Figure 1:**
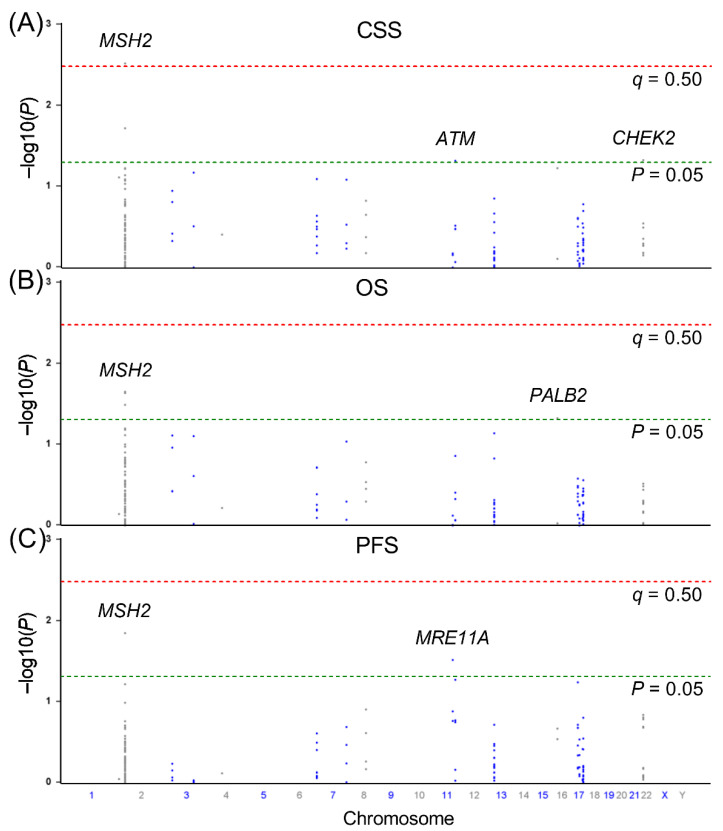
Manhattan plots of the associations (−log10 (*p*) Values; *Y*-axis) between 167 single nucleotide polymorphisms (SNPs; *X*-axis by chromosome positions) in 18 DNA repair pathway genes and (**A**) cancer-specific survival, (**B**) overall survival, and (**C**) progression-free survival among patients with prostate cancer treated with androgen deprivation therapy. Labelled genes contain associated SNPs with *p* < 0.05. The red horizontal line represents the suggestive significance threshold of *q* = 0.50, and the green horizontal line represents the nominal significance threshold of *p* = 0.05.

**Figure 2:**
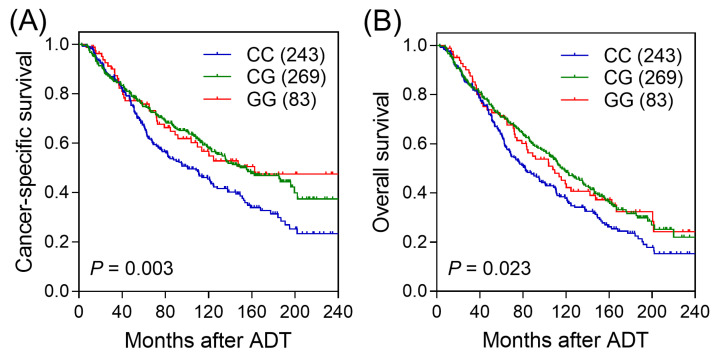
Kaplan-Meier curves of (**A**) cancer-specific survival and (**B**) overall survival after androgen deprivation therapy (ADT) for *MSH2* rs1400633 genotypes. Values in brackets represent the number of patients.

Univariate survival analysis on the association of 167 SNPs in the DDR pathway genes with CSS, OS, and PFS is summarised in Figure 1. Four SNPs (rs1400633 and rs4953519 in *MSH2*, rs4987981 in *ATM*, and rs9620817 in *CHEK2*) were associated with CSS, four SNPs (rs1400633, rs2303425, and rs13383462 in *MSH2*, and rs249954 in *PALB2*) were associated with OS, and two SNPs (rs11903353 in *MSH2* and rs496797 in *MRE11A*) were associated with PFS (*p* < 0.05). After applying multiple testing correction (*q* < 0.50), only *MSH2* rs1400633 was significantly associated with CSS. Each additional rs1400633 minor allele G was associated with a 22% decreased risk of cancer-specific mortality (HR = 0.78, 95% confidence interval [CI] = 0.65–0.92, *p* = 0.003, *q* = 0.492) (Table 2 and Figure 2A). This association remained significant after an adjustment for clinical predictors and coincided with a 25% decreased risk of cancer-specific mortality (adjusted HR = 0.75, 95% CI = 0.63–0.90, *p* = 0.002). Interestingly, *MSH2* rs1400633 was also associated with OS in univariate and multivariate analyses (adjusted HR = 0.84, 95% CI = 0.72–0.98, *p* = 0.029) (Table 2 and Figure 2B).

**Figure 3:**
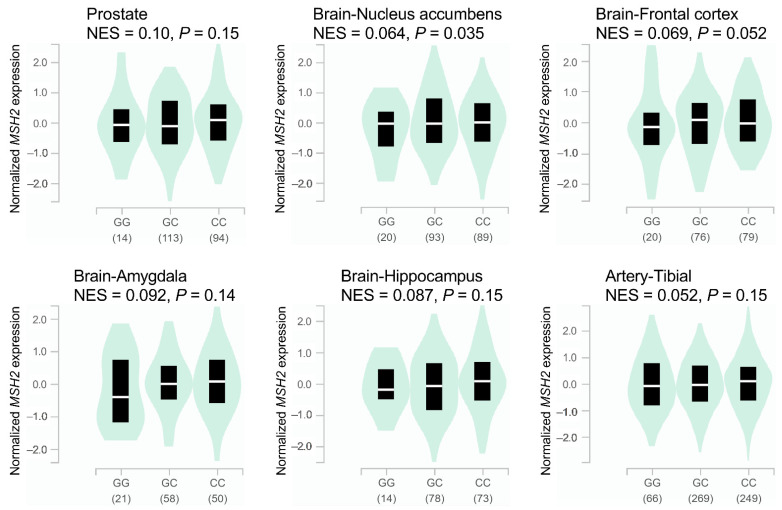
Correlation between rs1400633 genotypes and *MSH2* expression in different organs. Values were calculated using prostate, brain nucleus accumbens, frontal cortex, amygdala, hippocampus, and tibial artery tissue data from the Genotype-Tissue Expression project. Values in brackets represent the number of samples. NES, normalized effect size.

The HaploReg analysis demonstrated that rs1400633 and several linked SNPs in linkage disequilibrium (*r*^2^ > 0.8) were located in functional promoters/enhancers with active histone marks and DNase hypersensitive sites, which made them more accessible to transcription factors in multiple tissues (Table 3). Additionally, these SNPs may alter the motif of some transcription regulators, which could affect *MSH2* expression via alterations to chromatin remodelling during transcription. According to the GTEx database, rs1400633 risk allele C was associated with a significant increase in *MSH2* mRNA expression in the brain nucleus accumbens (normalized effect size [NES] = 0.064, *p* = 0.035), and the same trend, positive NES, has also been observed in the prostate (NES = 0.10, *p* = 0.15) and several other types of tissues (Figure 3).

**Figure 4:**
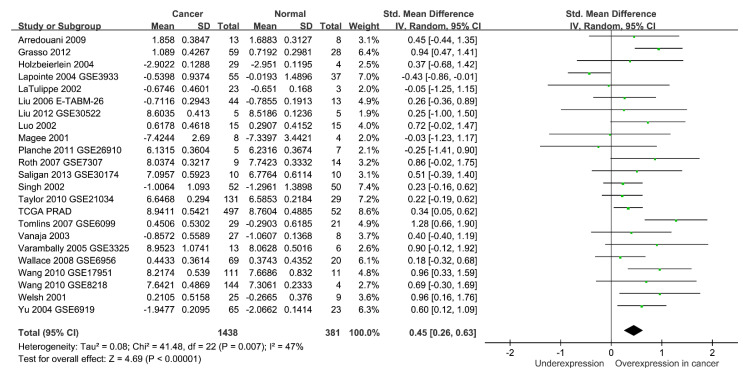
Meta-analysis of *MSH2* expression between normal and prostate cancer tissues in 23 independent studies. *MSH2* expression is significantly upregulated in prostate cancer. Abbreviations: TCGA PRAD, The Cancer Genome Atlas Prostate Adenocarcinoma; SD, standard deviation; IV, inverse variance; CI, confidence interval; Std, standardized; df, degrees of freedom.

**Figure 5:**
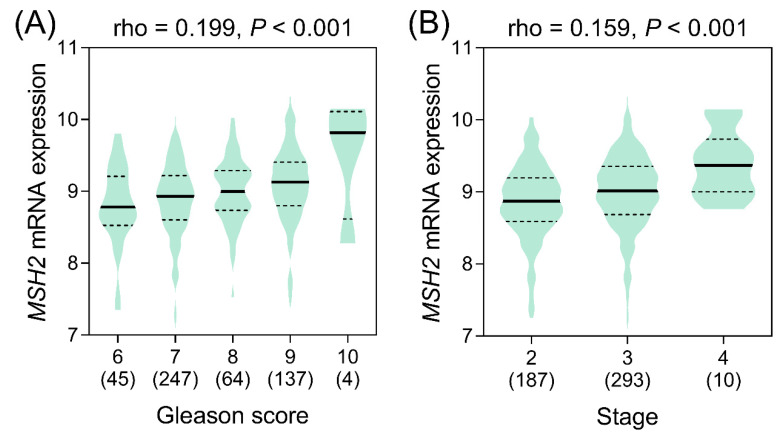
Correlation of *MSH2* expression with (**A**) Gleason score and (**B**) tumour stage in The Cancer Genome Atlas Prostate Adenocarcinoma dataset. Rho, Spearman’s rank correlation coefficient. Values in brackets represent the number of samples.

**Figure 6:**
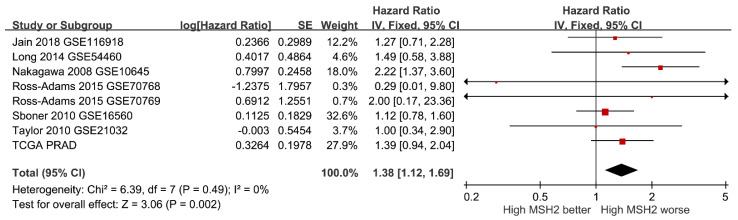
Meta-analysis of eight studies evaluating the prognostic role of *MSH2* in prostate cancer. SE, standard error; IV, inverse variance; CI, confidence interval; TCGA PRAD, The Cancer Genome Atlas Prostate Adenocarcinoma.

Finally, we assessed the clinical relevance of *MSH2* expression in prostate cancer. A meta-analysis of 23 studies that included 1438 prostate cancer and 381 adjacent normal samples demonstrated elevated *MSH2* expressions in prostate cancer (standardised mean difference = 0.45, 95% CI = 0.26–0.63, *p* < 0.001) (Figure 4). High *MSH2* expression was significantly associated with a higher Gleason score and a more advanced tumour stage in the TCGA PRAD dataset (*p* < 0.001) (Figure 5). Furthermore, a meta-analysis of eight studies comprising 2071 patients suggested that a high expression of *MSH2* was associated with a poor prognosis for prostate cancer (HR = 1.38, 95% CI = 1.12–1.69, *p* = 0.002) (Figure 6). These pooled results indicated that high *MSH2* expression might play an important role in the development and progression of prostate cancer.

## 4. Discussion

In the present study, we explored the associations between 167 htSNPs of 18 DDR pathway genes and the efficacy of ADT. We report that *MSH2* rs1400633 C > G was independently associated with both the CSS and OS of patients with advanced prostate cancer subjected to ADT. The bioinformatic analyses based on the GTEx database suggested that the risk allele C of rs1400633 was related to the increased *MSH2* expression. Furthermore, a meta-analysis of 31 studies revealed that *MSH2* expression was elevated in prostate cancer tissues and was significantly associated with worse patient prognosis.

The risk variant rs1400633 and its linked SNPs were found to be located in a regulatory region. Using a multivariate hidden Markov model to identify chromatin states from multiple histone modifications according to the HaploReg database, the region was classified as a functional promoter/enhancer in trophoblast, mesenchymal stem, placenta, lymphoblastoid, and mammary epithelial cells. Therefore, rs1400633 may affect gene expression by regulating the accessibility of DNA to the transcriptional machinery and transcription factors. Additionally, rs1400633 could disrupt the binding of some important transcription regulators, including the pancreas associated transcription factor 1a (PTF1A) and paired box 5 (PAX5). Paired box transcription factors play critical roles in early development, and the dysregulation of these genes is thought to contribute to neoplastic transformation, such as rhabdomyosarcoma and lymphoma [37]. Upregulation of PAX5 was found to induce the forkhead box P4 axis and promote tumorigenesis in prostate cancer [38]. Consistent with that, we found that the risk allele C of rs1400633 was associated with a significant increase in *MSH2* mRNA expression in the brain nucleus accumbens and exhibited a similar trend in prostate tissues.

MSH2 is a core protein in the mammalian DNA mismatch repair pathway that is responsible for recognising and removing base pair mismatches introduced by replication errors. MSH2 forms two heterodimers with MSH3 or MSH6, and these protein complexes are responsible for the recognition of single nucleotide and small insertion/deletion mismatches [39]. Upon mismatch recognition, the complexes recruit the MLH1/PMS2 heterodimer to nick the 5′ of the mismatches via PMS2 endonuclease activity. Then, exonuclease 1 removes the mismatch, DNA polymerase δ 1 resynthesizes the strand, and DNA ligase 1 seals the nick, thereby completing the DNA mismatch repair [40,41]. Given the role of MSH2 in DNA repair, mutations in *MSH2* are likely to affect this process, lead to genomic instability, and drive tumorigenesis. Conflicting data on the protein expression of MSH2 in prostate cancer exist; while some studies have suggested a link between loss of MSH2 expression and aggressive prostate cancer features [42,43], others have associated overexpression of MSH2 with prostate cancer progression and poor patient prognosis [44,45,46]. MSH2 deficiency has been described in 1.2–8.0% of prostate cancer patients and is more frequent in tumours with Gleason scores of 9–10 [42,43]. In contrast, several studies have demonstrated elevated MSH2 in prostate tumours, together with a higher Gleason score, as well as worse disease-free rates and overall survival [44,45,46], which is in line with our findings. Given that these studies have relied on protein expression in antibody-based approaches, the discrepancies might be associated with the methodology, sample size, and the specificity and sensitivity of the antibodies. Furthermore, the differentiation of functionally normal protein from abnormal protein remains difficult using immunohistochemical staining. Besides prostate cancer, MSH2 is often overexpressed in melanoma, gastric cancer, and oral squamous cell carcinoma, where it correlates with increased tumour aggressiveness and poor patient prognosis [47,48,49]. Increased expression of other DNA mismatch repair proteins, such as MSH6, MLH1, and PMS1, has also been observed in multiple cancers [50]. This phenomenon can be explained by the higher proliferation rate of cancer cells and their stronger need to correct mutations and DNA damage during DNA replication.

Two potential implications may translate our findings into clinical practice. First, genotyping patients prior to ADT could help identify patients at high risk of therapeutic failure, and these patients may benefit from a more aggressive treatment strategy, such as the addition of novel drugs to ADT. For example, the clinical trials supported the combination of docetaxel and ADT in the first-line treatment of metastatic castration-sensitive prostate cancer [51,52]. Second, this study provides evidence that the genetic variants in DDR pathways may influence the efficacy of ADT. Recently, poly (ADP-ribose) polymerase (PARP) inhibitors have been tried for the treatment of metastatic castration-resistant prostate cancer [53,54]. Because DDR-deficient cancers are unable to repair DNA double-strand breaks, they are sensitive to PARP inhibition, explaining the rationale for this approach. Moreover, androgen signalling has been found to regulate the expression and function of DDR genes in prostate cancer cells, which may justify the synergistic combination of ADT and PARP inhibitors [55,56]. Together, the above preclinical and clinical evidence offers a potential biological explanation for the observed association between *MSH2* rs1400633 and prostate cancer progression; however, further experiments are required to identify the exact mechanism.

The major strength of this study is the availability of complete clinical information to adjust for confounding variables. Additional strength stems from our comprehensive evaluations of genetic variants in DDR pathways using the haplotype tagging approach. However, the results reported here are limited by the modest sample size and homogeneous Taiwanese study population, which reduces the ability to extrapolate our findings to other ethnic groups. Additional fine-mapping to narrow down the causal variants, followed by validation in independent and functional studies, is necessary.

## 5. Conclusions

The consistent risk associations across our genetic analyses and independent gene expression studies suggest that the genes involved in DDR, such as *MSH2,* could have a prognostic function in prostate cancer survival. For patients receiving ADT, *MSH2* rs1400633 may serve as a novel prognostic biomarker or a clinical decision-making indicator. Future functional studies should elucidate the biological mechanisms defining how genetic variants can affect prostate cancer mortality.

## Figures and Tables

**Table 1:** Clinicopathological characteristics of the study population.

Characteristics		CSS ^a^		OS ^a^		PFS ^a^	
		HR (95% CI)	*p*	HR (95% CI)	*p*	HR (95% CI)	*p*
Total, *n* (%)	630	314 (49.8)		414 (65.7)		518 (82.6)	
Age at diagnosis, years							
Median (IQR)	73 (67–79)	1.012 (0.999–1.026)	0.078	1.029 (1.016–1.041)	<0.001	0.980 (0.970–0.990)	<0.001
PSA at ADT initiation, ng/mL							
Median (IQR)	34.5 (11.25–129)	1.000 (1.000–1.000)	<0.001	1.000 (1.000–1.000)	<0.001	1.000 (1.000–1.000)	0.001
PSA nadir, ng/mL							
Median (IQR)	0.14 (0.01–1.16)	1.002 (1.001–1.002)	<0.001	1.002 (1.001–1.002)	<0.001	1.001 (1.000–1.001)	0.019
Time to PSA nadir, months							
Median (IQR)	11 (5–20)	0.972 (0.962–0.981)	<0.001	0.982 (0.975–0.990)	<0.001	0.956 (0.948–0.963)	<0.001
Clinical stage at diagnosis							
T1/T2, *n* (%)	187 (29.9)	Reference		Reference		Reference	
T3/T4/N1, *n* (%)	205 (32.8)	1.080 (0.785–1.488)	0.636	1.065 (0.818–1.386)	0.642	0.860 (0.686–1.078)	0.190
M1, *n* (%)	233 (37.3)	3.096 (2.334–4.106)	<0.001	2.508 (1.968–3.194)	<0.001	1.406 (1.136–1.739)	0.002
Gleason score at diagnosis							
2–6, *n* (%)	188 (30.6)	Reference		Reference		Reference	
7, *n* (%)	194 (31.6)	1.063 (0.783–1.443)	0.694	1.064 (0.820–1.381)	0.640	1.162 (0.929–1.455)	0.189
8–10, *n* (%)	232 (37.8)	2.144 (1.629–2.820)	<0.001	1.957 (1.542–2.484)	<0.001	1.483 (1.194–1.841)	<0.001

Abbreviations: CSS, cancer-specific survival; OS, overall survival; PFS, progression-free survival; IQR, interquartile range; PSA, prostate-specific antigen. ^a^ With a median follow-up of 165.8 months. Subtotals do not sum to 630 due to missing data.

**Table 2:** Association of *MSH2* rs1400633 with cancer-specific survival and overall survival in prostate cancer patients receiving androgen deprivation therapy.

Genotype	Frequency	CSS				OS			
		HR (95% CI)	*p*	HR (95% CI) ^a^	*p* ^a^	HR (95% CI)	*p*	HR (95% CI) ^a^	*p* ^a^
CC/CG/GG	244/269/84	0.78 (0.65–0.92)	0.003	0.75 (0.63–0.90)	0.002	0.84 (0.73–0.98)	0.023	0.84 (0.72–0.98)	0.029

Abbreviations: CSS, cancer-specific survival; OS, overall survival; HR, hazard ratio; CI, confidence interval. ^a^ Adjustment for age, stage, Gleason score at diagnosis, prostate-specific antigen at androgen deprivation therapy initiation, prostate-specific antigen nadir, and time to prostate-specific antigen nadir.

**Table 3:** Functional annotation of *MSH2* rs1400633 and its linked variants.

Variant	Position	LD (*r*^2^)	Reference Allele	Alternate Allele	ASN Frquency	Promoter Histone Marks	Enhancer Histone Marks	DNase	Proteins Bound	Motifs Changed
rs1882450	47647464	0.82	G	C	0.64					2 altered motifs
rs2091750	47647822	0.8	T	G	0.65					5 altered motifs
rs1400633	47656863	1	G	C	0.65		4 tissues	5 tissues	3 bound proteins	2 altered motifs
rs2969775	47658337	1	G	T	0.65		2 tissues			7 altered motifs
rs2969774	47661684	0.94	T	C	0.65		2 tissues			2 altered motifs
rs2705766	47663192	0.95	T	C	0.66	2 tissues	3 tissues	1 tissue		2 altered motifs
rs2705767	47664866	0.92	G	A	0.65		2 tissues		1 bound protein	5 altered motifs
rs2944783	47665860	0.92	G	C	0.65					8 altered motifs
rs2969772	47667333	0.9	A	G	0.65					

Abbreviations: LD, linkage disequilibrium; ASN, Asia.

## Data Availability

The data presented in this study are available on request from the corresponding author.
